# Knowledge, attitudes and practices regarding antimicrobial usage, spread and resistance emergence in commercial poultry farms of Rajshahi district in Bangladesh

**DOI:** 10.1371/journal.pone.0275856

**Published:** 2022-11-15

**Authors:** Md. Zohurul Islam, Md. Saiful Islam, Lakshmi Rani Kundu, Ayesha Ahmed, Kamrul Hsan, Shahina Pardhan, Robin Driscoll, Md. Sharif Hossain, Md. Mahfuz Hossain

**Affiliations:** 1 Department of Public Health and Informatics, Jahangirnagar University, Savar, Dhaka, Bangladesh; 2 Centre for Advanced Research Excellence in Public Health, Savar, Dhaka, Bangladesh; 3 Humanitarian Response Organization, Dhaka, Bangladesh; 4 Vision and Eye Research Institute, School of Medicine, Anglia Ruskin University, Cambridge, United Kingdom; Guru Angad Dev Veterinary and Animal Sciences University, INDIA

## Abstract

**Background:**

Inappropriate and injudicious use of antimicrobials in broiler and layer farms has become a common practice in lower and middle-income countries including Bangladesh. This study aimed to assess poultry farmers’ knowledge, attitude, and practices regarding antimicrobial usage (AMU), and their beliefs in factors that affect antimicrobial resistance (AMR) spread and emergence in humans through commercial poultry farms in Bangladesh.

**Methods:**

A cross-sectional study was conducted among 204 farmers (95.6% male; mean_age_ = 35.14 ± 10.25 years) in the Rajshahi district of Bangladesh who were recruited from three upazilas (sub-districts) through a multistage sampling technique. Data were collected from June to November 2021 via face-to-face interviews using a semi-structured questionnaire.

**Results:**

The proportion of farmers who reported having received information regarding AMU from veterinarians was higher in layer compared to broiler farms (65.9% vs. 44.9%, *p* < 0.001). A higher proportion of layer compared to broiler farmers believed that antimicrobial residues and pathogens in poultry can pass to humans through the consumption of contaminated eggs (28.1% vs. 5.8%, *p* < 0.05). The mean score of the farmers’ attitude towards addressing AMU was 4.49 (SD = 1.37) out of 7, with the higher score indicating a better attitude. The mean score of better attitudes towards addressing AMU was significantly higher among educated participants (bachelor’s or higher levels of education (*p* = 0.006). A higher proportion of layer (56.3%) farmers did not keep a record of AMU when compared to broiler farmers (37.7%) (*p* = 0.012). More broiler (50.7%) compared to layer (38.5%) farmers continued using the full dose of antimicrobials (*p* = 0.042). The most frequently used antimicrobials in broiler and layer poultry farms were Colistin (broiler vs layer: 73.9% vs. 86.75%; *p* = 0.024), and Ciprofloxacin (broiler vs. layer: 95.7% vs. 84.4%; *p* = 0.021). Farmers’ beliefs were significantly associated with the spread of AMR pathogens from contaminated eggs to humans (*p* < 0.001).

**Conclusions:**

The findings reflected that majority of farmers had inadequate knowledge of AMU, less knowledgeable beliefs aboutAMU, and inappropriate AMU (e.g., poor record keeping, incomplete doses) in chicken production systems. The government should ensure education or advisory services for poultry farmers on proper AMU, enforce current veterinary laws and regulations on antimicrobials, and implement AMU surveillance systems.

## Introduction

Rapid population growth and income are increasingly influencing the demand for meat and poultry products in many developing countries. Poultry meat production has increased significantly over the years in South and South East Asia [1,2], including poultry bearing in Bangladesh since 1990 [3].

There are about 150K commercial poultry (broiler and layer) farms in Bangladesh [4], with a minimum 50% of those being layer farms [5]. In Bangladesh, 37% of all protein from animal sources comes from poultry [6]. Two poultry producing systems exist in Bangladesh: commercial and backyard production. Approximately, 89% of households rear chickens with an average flock size of seven birds [6–8]. Commercial chicken production is divided into two categories: broiler and layer. Broiler farming raises chickens for meat, whereas layer farming raises hens for egg production; however, dysfunctional layer birds may also be sold for meat [9]. The prevalence of maladies is the most challenging problem for commercial chicken farmers [10]. As a result, commercial chicken production usually requires intensive animal husbandry procedures, such as antibiotic treatment and vaccines [11].

There are different needs for antimicrobial treatments: therapeutic and prophylactic [12]. Antimicrobials are often used in Bangladesh for both the treatment and prevention of chicken disease, but some farmers also use them for growth promotion and to enhance feed intake [13]. While the use of antimicrobials has led to a decrease in animal death and morbidity rates, antimicrobial abuse is regarded as one of the most serious global public health threats in this century [14,15]. Thus, the gradual emergence of antimicrobial resistance can lead to therapeutic failure for animals [16] and human ailments [17].

Antimicrobial-resistant infections affected by antimicrobial usage (AMU) in animals may be transmitted to people by direct contact, ingestion of meat and eggs, or indirectly via environmental routes [18]. Experts believe that the global use of antimicrobial agents in animals is double that compared to people, even though the underlying statistics from the veterinary sector supporting these estimates may be weak and inconsistent [19,20]. Currently, the health authority’s primary priority is to safeguard public health from any hazardous consequences of these veterinary medicines [21,22]. Modern animal production techniques in Bangladesh and many other countries are associated with the frequent use of antimicrobials, increasing the selection pressure on bacteria to become resistant [23]. In 2015, the global average consumption of antimicrobials per kilogram of chicken produced was estimated to be 148 mg/kg, and the worldwide AMU in animals for human consumption was predicted to be 63,000 tons per year, whereas, by 2030, the global AMU in livestock is expected to grow by about 70% [23]. The scope of antimicrobial use in animal production in Bangladesh is unclear [24], and statistics on national antimicrobial sales are scarce [25]. The Animal Feed Act of Bangladesh forbids all use of antibiotics in feed [26], however; the widespread sales of antimicrobials through feed and chick merchants and pharmaceutical company representatives [27] demonstrate Bangladesh’s lack of antimicrobial governance. The Bangladesh government issued a list of essential medications for human treatment in the National Drug Policy 2016 that should not be supplied “over the counter” [28]. Besides that, there are no laws on veterinary medication registration nor clear recommendations for the use of antimicrobials in food animals in Bangladesh [25,28]. Only registered veterinarians are permitted to give medication or conduct surgery under the Bangladesh Veterinary Practitioners Ordinance, 1982 [25,29]. According to the Drug Act of 1940, only registered pharmacists are permitted to offer antibiotics with a legal prescription. Controlling AMU and preventing its abuse in poultry is influenced by farmers’ compliance with antimicrobial standards and their perceptions of the implications of AMR development [30].

The World Health Organization’s Global Action Plan on Antimicrobial Resistance advised that AMUs should be monitored through surveillance and research to assist in preventing the development and spread of AMR infections in both animals and humans [31]. The present study was conducted to assess poultry farmers’ knowledge, attitude, and practices regarding AMU and to determine the factors that farmers believed were associated with pathways for AMR emergence and spread to humans through commercial poultry farms in Bangladesh.

## Materials and methods

### Study design and population

A cross-sectional study was conducted from June to November 2021. The study population were farmers involved in poultry production. A total of 219 commercial poultry (both layer and broiler) farmers were selected randomly for the study, with a final number of 204 taking part. The inclusion criteria of the participants included: (i) being a poultry farmer (either broiler or layer); (ii) being involved actively in chicken management on the visited farm; and (iii) being able to provide information about their farms. Participation was fully voluntary and uncompensated. The exclusion criteria were: (i) participants no longer operating or having no chickens at the time of the field visits; (ii) participants who were unable to provide information about their farms; and (iii) farms which were neither broiler nor layer were excluded from the current study.

### Study area

The study was conducted in the Rajshahi district located in the northwest part of Bangladesh under the Rajshahi division bordering India to the south. It is approximately 258 kilometers from the capital (Dhaka) of Bangladesh and located at a latitude of 24°07’-24°43’ north and a longitude of 88°17’-88°58’ east. The study area is surrounded by Naogaon district on the north; the West Bengal state of India, Kushtia district and the Ganges River on the south; Natore district on the east; and Nawabganj district on the west. At present, the Rajshahi district is one of the main districts in the country in terms of poultry production and also the main region supplying poultry to Rajshahi city. Farmers in the region are greatly involved in the raising of poultry, yet there is limited information on the utilization of antibiotics.

### Sample size determination

The sample size was calculated using the following formula:

$$n=\frac{z^{2}pq}{d^{2}}$$

Where, *z* = 1.96 at 5% level of significance and 7% acceptable margin of error (*d* = 0.07).

Since there was no similar study in this cohort in the study area, we consider the maximum sample proportion as 50%. So, the minimum required sample size calculated for this study was 196. 204 participants were recruited to ensure the strength of the study.

### Sampling procedure

**Fig 1:**
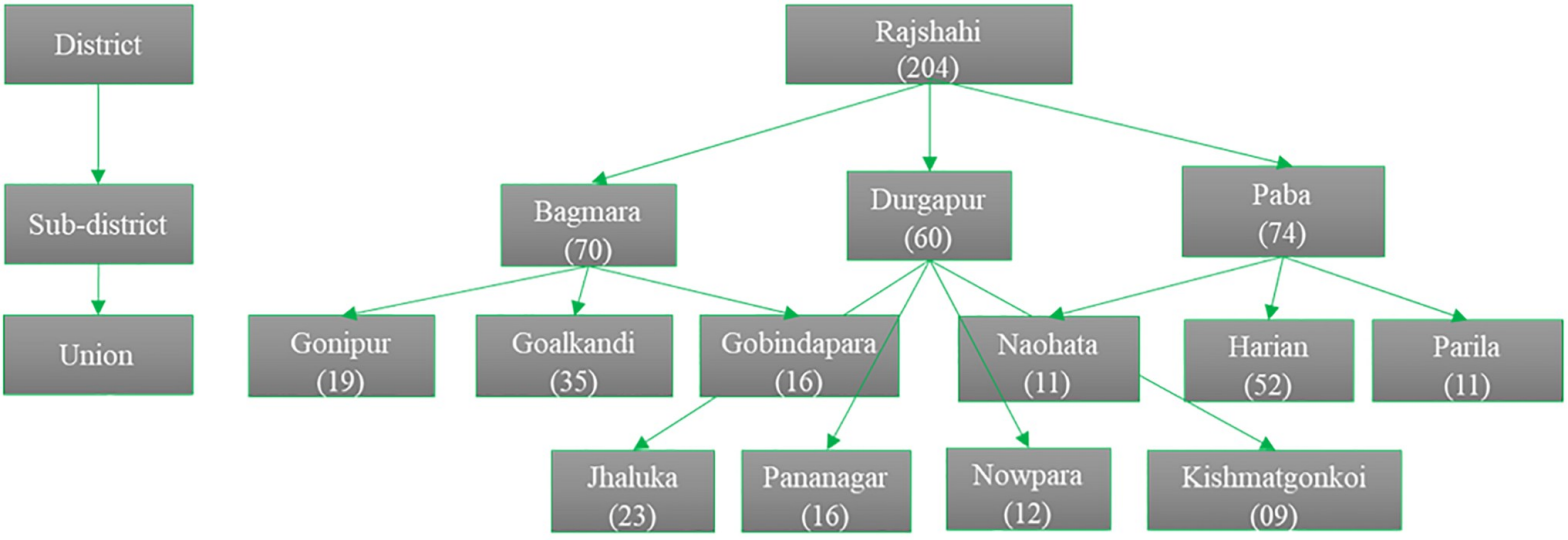
Flowchart of sampling procedure.

A multistage sampling technique was used to recruit study participants. A flow chart of the sampling procedure is included below (Fig 1):

### Data collection tools and techniques

Data were collected through face-to-face interviews using a semi-structured questionnaire. The questionnaire comprised of six sections that included: (i) socio-demographic characteristics and poultry farming information, (ii) knowledge about antimicrobial usage (AMU) in poultry farms, (iii) farmers’ beliefs regarding AMU, (iv) practices of AMU in poultry, (v) antimicrobials frequently used in poultry, and (vi) identification of pathways for the transmission of antimicrobial-resistant pathogens from poultry to humans.

#### Socio-demographic characteristics and poultry farming information

Questions related to socio-demographic characteristics and poultry farm information were asked during the interview, including age, sex, marital status, educational qualifications, religion, family income, main income source of family, number of sheds, number and type of chickens, age of chickens, type of production system, and experience in poultry farming.

### Knowledge about the use of AMU in poultry farms

To assess knowledge about antimicrobials usage, a total of 9 questions (e.g., ‘*Do you know or hear about antimicrobials usage*?; *Can antimicrobial-resistant pathogens in poultry be passed to humans*?’, see Table 3) were used. All questions were adopted from a previous study [32].

#### Farmers’ beliefs about AMU

The attitude section consisted of 7 questions, including 5 positive statements and 2 negative statements (e.g., ‘*If medications are given too often*, *their effectiveness may stop*; *Healthy chickens are less likely to get sick if given antimicrobials*’, see Table 3) [33]. All statements used a three-point Likert scale (e.g., disagree/neutral/agree). For positive statements, the responses were coded as ‘agree = 1’, and ‘disagree or neutral = 0”; and for negative statements, ‘disagree = 1’, ‘agree or neutral = 0’ [33]. The total score was obtained by summating the raw scores of each statement and ranged from 0–7, with a higher score indicating compliance with desirable AMU behavior.

#### Practices of AMU in poultry

This comprised 15 questions (e.g. ‘*Do you purchase antimicrobials according to prescription*?; *When a few chickens get sick*, *do you give antimicrobials to all chickens*?’ see Table 6). All questions were adopted from a previous study [29,32].

#### Pathways for the transmission of antimicrobial-resistant pathogens from poultry to humans

To assess farmers’ beliefs regarding the pathways for the transmission of antimicrobial-resistant pathogens from poultry to humans, 3-item questions were included (*contaminated poultry product*; *direct or indirect contact*; and *appearance of waste material in the environment*; see details in Table 7) [32].

### Data analysis

All statistical analyses were carried out using the Statistical Package for the Social Sciences (SPSS) version 25.0. Descriptive statistics (frequencies, percentages, means, standard deviations [SDs]) were computed. Bivariate analyses (e.g., Chi-square test, Fisher’s exact test, t-test, and Analysis of Variance [ANOVA]) were performed to determine the association between the dependent and independent variables as appropriate. All statistical analyses were conducted at 5% level of significance.

### Ethics

The study protocol was reviewed and approved by the Biosafety, Biosecurity, and Ethical Clearance Committee, Jahangirnagar University, Savar, Dhaka-1342, Bangladesh [Ref No: BBEC, JU/ M- 2022/ 2(3)]. Informed written consent was obtained from all the participants before data collection. The objectives of the research were explained to the participants, and they were informed that they could choose to participate (or not) in the study. The confidentiality of information and anonymity of the participants was strictly maintained.

## Results

### Socio-demographic and farming characteristics of the respondents

A total of 204 poultry farmers participated in the study, with a mean age of 35.14 (SD = 10.25) years. Most were males (95.6%; n = 195) and 87.3% (n = 178) were married (Table 1). 30.4% of the participants were educated to Honors level or higher (n = 62), and only a few respondents had no formal education (7.8%, n = 16). For the majority of participants (84.3%, n = 172), their income source was poultry farming; however, some participants also received income from different types of agriculture or business. With regards to the type of farm, 66.2% (n = 135) were layer farmers. The majority of participants (58.8%, n = 120) had fewer than 1500 chickens, and the mean age of the chickens was 170.5 (SD = 152.3) days. 55.9% (n = 114) of participants had less than one chicken shed. Just over half (50.5%, n = 103) had over 5 years of experience in poultry rearing. 52.5% of respondents (n = 107) were involved in continuous production processing.

**Table 1:** Socio-demographic and farming characteristics of broiler and layer farmers.

Variables	Overall N = 204	Broiler (69; 33.8%)	Layer (135; 66.2%)	*p*-value
	*n* (%)	*n* (%)	*n* (%)	
**Age (Mean± SD)**	35.14±10.25	33.8±10.0	35.8±10.3	0.169^§^
**Sex**				
Male	195 (95.6)	66 (95.7)	129 (95.6)	1.000^†^
Female	9 (4.4)	3 (4.3)	6 (4.4)	
**Educational qualification**				
No formal education	16 (7.8)	4 (5.8)	12 (8.9)	0.643^†^
Primary	21 (10.3)	10 (14.5)	11 (8.1)	
Secondary	60 (29.4)	19 (27.5)	41 (30.4)	
Intermediate	45 (22.1)	16 (23.2)	29 (21.5)	
Honors or above	62 (30.4)	20 (29.0)	42 (31.1)	
**Marital Status**				
Married	178 (87.3)	56 (81.2)	122 (90.4)	0.132^†^
Unmarried	24 (11.8)	12 (17.4)	12 (8.9)	
Divorced	2 (1.0)	1 (1.4)	1 (.7)	
**Family Income**				
<15,000 BDT	30 (14.7)	10 (14.5)	20 (14.8)	0.786^‡^
15,000–30,000 BDT	124 (60.8)	44 (63.8)	80 (59.3)	
>30,000 BDT	50 (24.5)	15 (21.7)	35 (25.9)	
**Income source**				
Poultry farming	172 (84.3)	56 (81.2)	116 (85.9)	0.376^‡^
Fishing	3 (1.5)	0 (0.0)	3 (2.2)	0.552^†^
Agriculture	34 (16.7)	12 (17.4)	22 (16.3)	0.843^‡^
Business	36 (17.6)	10 (14.5)	26 (19.3)	0.398^‡^
**Number of chickens**				
≤1,500	120 (58.8)	51 (73.9)	69 (51.1)	**.002** ^†^
1,501–3,000	62 (30.4)	16 (23.2)	46 (34.1)	
>3,000	22 (10.8)	2 (2.9)	20 (14.8)	
**Number of sheds**				
1	114 (55.9)	48 (69.6)	66 (48.9)	**.005** ^‡^
>1	90 (44.1)	21 (30.4)	69 (51.1)	
**Chicken age (Mean± SD)**	170.5±152.3	34.2±25.7	240.1±142.7	**<0.001** ^§^
**Production system**				
All in all out	93 (45.6)	64 (92.8)	29 (21.5)	**<0.001** ^†^
Continuous	107 (52.5)	5 (7.2)	102 (75.6)	
Both	4 (2.0)	0 (.0)	4 (3.0)	
**Experience in poultry farming**				
<6 months	8 (3.9)	5 (7.2)	3 (2.2)	**.002** ^†^
6–12 months	19 (9.3)	10 (14.5)	9 (6.7)	
1–5 years	74 (36.3)	31 (44.9)	43 (31.9)	
5 years	103 (50.5)	23 (33.3)	80 (59.3)	

***Note***:

^†^Fisher’s Exact test;

^‡^Chi-square test;

^§^t-test;

BDT = Bangladeshi Taka.

### Association of socio-demographic and farming characteristics with farmers

Bivariate analysis showed no significant association between socio-demographic characteristics and farmers (broiler and layers). However, the farmers were significantly associated with farming characteristics including number of chickens (*p* = 0.002), number of sheds (*p* = 0.005), chicken age (*p* < 0.05), production system (*p* < 0.05), and experience in poultry farming (*p* = 0.002) (Table 2).

**Table 2:** Distribution of knowledge/awareness about antimicrobial usage among broiler and layer farmers.

Variables	Overall N = 204	*Broiler*	*Layer*	*p*-value
	*n* (%)	*n* (%)	*n* (%)	
**Know/heard about antimicrobial usage**				
Yes	182 (89.2)	61 (88.4)	121 (89.6)	0.790
No	22 (10.8)	8 (11.6)	14 (10.4)	
**Source of information about antimicrobial usage**				
Veterinarian	120 (58.8)	31 (44.9)	89 (65.9)	<**0.001**
Poultry traders	58 (28.4)	31 (44.9)	27 (20.0)	
Pharmaceutical representative	26 (12.7)	7 (10.1)	19 (14.1)	
**Know antimicrobials to be used**				
To treat infections in chickens	98 (48.0)	33 (47.8)	65 (48.1)	0.847
To prevent infections in chickens	47 (23.0)	16 (23.2)	31 (23.0)	
To promote growth in chickens	11 (5.4)	5 (7.2)	6 (4.4)	
I don’t know	48 (23.5)	15 (21.7)	33 (24.4)	
**Antimicrobial abuse is when**				
Administered under-dose	91 (44.6)	37 (53.6)	54 (40.0)	0.129^†^
Administered over-dose	57 (27.9)	16 (23.2)	41 (30.4)	
Administered in normal dose	3 (1.5)	2 (2.9)	1 (.7)	
I don’t know	53 (26.0)	14 (20.3)	39 (28.9)	
**Knowledge about antimicrobial resistance**				
Yes	123 (60.3)	41 (59.4)	82 (60.7)	0.855
No	81 (39.7)	28 (40.6)	53 (39.3)	
**Effect of antimicrobial resistance in chickens**				
Non-response to microbial infection treatment	82 (40.2)	28 (40.6)	54 (40.0)	0.996
Extra costs on the treatment of microbial infection	36 (17.6)	12 (17.4)	24 (17.8)	
I don’t know	86 (42.2)	29 (42.0)	57 (42.2)	
**Antimicrobial-resistant pathogens in poultry can be passed to humans**				
Yes	122 (59.8)	41 (59.4)	81 (60.0)	0.809
No	18 (8.8)	5 (7.2)	13 (9.6)	
I don’t know	64 (31.4)	23 (33.3)	41 (30.4)	
**Antimicrobial residues and pathogens in poultry can pass to humans through** *				
Consumption of contaminated eggs	42 (20.6)	4 (5.8)	38 (28.1)	**<0.001** ^†^
Consumption of contaminated meat	78 (38.2)	31 (44.9)	47 (34.8)	0.160
Contacts of workers/keepers with birds	18 (8.8)	8 (11.6)	10 (7.4)	0.319
I don’t know	79 (38.7)	26 (37.7)	53 (39.3)	0.827
**Effects of antimicrobial resistance in humans**				
Non-response to microbial infection treatment	56 (27.5)	20 (29.0)	36 (26.7)	0.939^†^
Extra costs on the treatment of microbial infection	8 (3.9)	2 (2.9)	6 (4.4)	
Longer duration of illness and treatment	22 (10.8)	8 (11.6)	14 (10.4)	
I don’t know	118 (57.8)	39 (56.5)	79 (58.5)	

***Note***:

^†^Fisher’s Exact test;

*Multiple responses.

### Distribution of knowledge about antimicrobials usage among farmers

Knowledge about AMU among farmers and their association are presented in Table 3. Nearly 45% broiler (n = 31) and 65.9% layer (n = 89) farmers received their information from veterinarians (*p* < 0.001). A low number of broiler farmers (5.8%, n = 4) compared to layer farmers (28.1%, n = 38) believed that antimicrobial residues and pathogens in poultry can pass to humans through the consumption of contaminated eggs (*p* < 0.05).

**Table 3:** Farmers’ attitude towards antimicrobial usage (AMU).

Statements	Disagree	Neutral	Agree
	*n* (%)	*n* (%)	*n* (%)
If medications are given too often, their effectiveness may stop	14 (6.9)	52 (25.5)	138 (67.6)
Healthy chickens are less likely to get sick if given antimicrobials	34 (16.7)	22 (10.8)	148 (72.5)
Healthy chickens can be given antimicrobials to help them grow faster and increase egg production	76 (37.3)	23 (11.3)	105 (51.3)
It is important to consult a veterinarian before giving antimicrobials to animals	8 (3.9)	9 (4.4)	187 (91.7)
The use of antibiotics can be reduced by using vaccines	31 (15.2)	30 (14.7)	143 (70.1)
After using antibiotics in poultry, we should wait for a while to use the meat/eggs produced from it	19 (9.3)	19 (9.3)	166 (81.4)
Antimicrobials can be harmful to human health	17 (8.3)	16 (7.8)	171 (83.8)

### Farmers’ beliefs regarding AMU

The distribution of farmers’ beliefs about AMU is presented in Table 4. The mean score of the beliefs was 4.49 (SD = 1.37) out of 7, the higher score indicating more desirable attitudes towards AMU. The mean attitude score was significantly higher among participants with higher levels of education (Bachelor’s or above) compared to those with no formal education (4.9 ± 1.3 vs. 3.8 ± 1.5; *p* = 0.006) (Table 5).

**Table 4:** Distribution of farmers’ demographic, farming characteristics, and attitudes towards AMU.

Variables	Attitudes		t/F	p-value
	Mean	(SD)		
**Age**	—	—	-.121	.085*
**Sex**				
Male	4.5	(1.4)	1.189	.277
Female	4.0	(1.3)		
**Educational Qualification**				
No formal education	3.8	(1.5)	3.694	**.006**
Primary	4.3	(1.1)		
Secondary	4.2	(1.4)		
Intermediate	4.7	(1.3)		
Bachelor or above	4.9	(1.3)		
**Marital Status**				
Married	4.5	(1.4)	.640	.528
Unmarried	4.6	(1.3)		
Divorced	5.5	(.7)		
**Family Income**				
<15,000 BDT	4.2	(1.6)	.804	.449
15,000–30,000 BDT	4.5	(1.3)		
>30,000 BDT	4.6	(1.5)		
**Number of chickens**				
≤1,500	4.4	(1.4)	2.095	.126
1,501–3,000	4.4	(1.3)		
>3,000	5.0	(.9)		
**Production system**				
All in all out	4.5	(1.4)	1.749	.177
Continuous	4.5	(1.3)		
Both	3.3	(.5)		
**Experience in poultry farming**				
<6 months	4.5	(1.3)	.431	.731
6–12 months	4.7	(.7)		
1–5 years	4.4	(1.5)		
5 years	4.5	(1.4)		
**Poultry farm types**				
Broiler	4.6	(1.5)	.661	.417
Layer	4.4	(1.3)		

***Note***:

*Pearson’s correlation test.

**Table 5:** Practices of AMU among farmers.

Variables	Overall N = 204	Broiler	Layer	*p*-value
	*n* (%)	*n* %	*n* %	
**Purchased antimicrobials according to prescription**				
Always	171 (83.8)	58 (84.1)	113 (83.7)	.059^†^
Sometimes	28 (13.7)	7 (10.1)	21 (15.6)	
Never	5 (2.5)	4 (5.8)	1 (.7)	
**Purchased antimicrobials from** *				
Veterinary drug shops	149 (73.0)	50 (72.5)	99 (73.3)	.895
Human drug shops	1 (.5)	1 (1.4)	0 (.0)	.338^†^
Poultry chick & feed traders	55 (27.0)	18 (26.1)	37 (27.4)	.841
**Administered antimicrobials to chickens by**				
Animal health officials	102 (50.0)	33 (47.8)	69 (51.1)	.448^†^
Self-administer	99 (48.5)	34 (49.3)	65 (48.1)	
Others	3 (1.5)	2 (2.9)	1 (.7)	
**Used antimicrobials on sick chickens**				
As indicated on leaflets	90 (44.1)	30 (43.5)	60 (44.4)	.113^†^
A single dose, once recovered	15 (7.4)	5 (7.2)	10 (7.4)	
Daily single dose until recovered	96 (47.1)	31 (44.9)	65 (48.1)	
Others	3 (1.5)	3 (4.3)	0 (.0)	
**Determined dosage before used**				
From instructions on the label	150 (73.5)	48 (69.6)	102 (75.6)	0.590
Arbitrary	20 (9.8)	7 (10.1)	13 (9.6)	
Others	34 (16.7)	14 (20.3)	20 (14.8)	
**When a few chickens get sick, give antimicrobials to all chickens**				
Always	176 (86.3)	58 (84.1)	118 (87.4)	.714^†^
Sometimes	19 (9.3)	7 (10.1)	12 (8.9)	
Never	9 (4.4)	4 (5.8)	5 (3.7)	
**The habit of using more than one antimicrobial together**				
Multiple antimicrobials together	82 (40.2)	21 (30.4)	61 (45.2)	.126
Only one type of antimicrobial	21 (10.3)	8 (11.6)	13 (9.6)	
Different antimicrobial for different treatment	101 (49.5)	40 (58.0)	61 (45.2)	
**Route of administered dose** *				
Water	203 (99.5)	69 (100.0)	134 (99.3)	1.000^†^
Feed	38 (18.6)	14 (20.3)	24 (17.8)	.663
Injection	3 (1.5)	0 (.0)	3 (2.2)	.552^†^
**Purposes for antimicrobial usage**				
Therapeutic	75 (36.8)	27 (39.1)	48 (35.6)	.482^†^
Prophylactic	31 (15.2)	7 (10.1)	24 (17.8)	
Growth development	1 (.5)	0 (.0)	1 (.7)	
Therapeutic and prophylactic	97 (47.5)	35 (50.7)	62 (45.9)	
**Frequency of antimicrobial dose**				
Once daily	75 (36.8)	24 (34.8)	51 (37.8)	.875
Twice daily	51 (25.0)	17 (24.6)	34 (25.2)	
Three times	78 (38.2)	28 (40.6)	50 (37.0)	
**Usage of the same dose**				
Yes	169 (82.8)	61 (88.4)	108 (80.0)	.132
No	35 (17.2)	8 (11.6)	27 (20.0)	
**Record book on antimicrobial usage**				
Yes	102 (50.0)	43 (62.3)	59 (43.7)	**.012**
No	102 (50.0)	26 (37.7)	76 (56.3)	
**Stop using antimicrobials before completing the full dose**				
Always	32 (15.7)	5 (7.2)	27 (20.0)	**.042**
Sometimes	85 (41.7)	29 (42.0)	56 (41.5)	
Never	87 (42.6)	35 (50.7)	52 (38.5)	
**Observed antimicrobials withdrawal periods**				
Yes	132 (64.7)	47 (68.1)	85 (63.0)	.466
No	72 (35.3)	22 (31.9)	50 (37.0)	
**Stop using antimicrobials before the sale**				
One month before selling chicken/eggs	16 (7.8)	2 (2.9)	14 (10.4)	.067^†^
Two weeks before selling chicken/eggs	8 (3.9)	3 (4.3)	5 (3.7)	
One week before selling chicken/eggs	50 (24.5)	12 (17.4)	38 (28.1)	
Until sell of chicken/eggs	49 (24.0)	22 (31.9)	27 (20.0)	
Others (Didn’t stop)	81 (39.7)	30 (43.5)	51 (37.8)	

***Note***:

^†^Fisher’s Exact test;

*Multiple responses.

### Distribution of practices of AMU among farmers

The distribution of practices of AMU among farmers can be seen in Table 6. A higher proportion of layer (56.3%, n = 76) and lower proportion of broiler (37.7%, n = 26) farmers did not keep any records of AMU (*p* = 0.012). In addition, a higher proportion of broiler (50.7%, n = 35) compared to layer (38.5%, n = 52) farmers always completed a full dose of antimicrobials (*p* = 0.042).

**Table 6:** Frequently used antimicrobials among broiler and layer farmers.

Variables	Overall N = 204	Broiler	Layer	*p*-value
	*n* (%)	*n* %	*n* %	
**Colistin**				
Yes	168 (82.4)	51 (73.9)	117 (86.7)	**.024**
No	36 (17.6)	18 (26.1)	18 (13.3)	
**Ciprofloxacin**				
Yes	180 (88.2)	66 (95.7)	114 (84.4)	**.021** ^†^
No	24 (11.8)	3 (4.3)	21 (15.6)	
**Tylosin**				
Yes	131 (64.2)	43 (62.3)	88 (65.2)	.686
No	73 (35.8)	26 (37.7)	47 (34.8)	
**Neomycin**				
Yes	123 (60.3)	44 (63.8)	79 (58.5)	.468
No	81 (39.7)	25 (36.2)	56 (41.5)	
**Amoxicillin**				
Yes	143 (70.1)	47 (68.1)	96 (71.1)	.658
No	61 (29.9)	22 (31.9)	39 (28.9)	
**Trimethoprim**				
Yes	50 (24.5)	15 (21.7)	35 (25.9)	.511
No	154 (75.5)	54 (78.3)	100 (74.1)	
**Sulphonamides**				
Yes	55 (27.0)	23 (33.3)	32 (23.7)	.143
No	149 (73.0)	46 (66.7)	103 (76.3)	
**Tiamulinok**				
Yes	78 (38.2)	28 (40.6)	50 (37.0)	.622
No	126 (61.8)	41 (59.4)	85 (63.0)	
**Penicillin**				
Yes	43 (21.1)	19 (27.5)	24 (17.8)	.106
No	161 (78.9)	50 (72.5)	111 (82.2)	
**Erythromycin**				
Yes	96 (47.1)	31 (44.9)	65 (48.1)	.663
No	108 (52.9)	38 (55.1)	70 (51.9)	
**Streptomycin**				
Yes	53 (26.0)	16 (23.2)	37 (27.4)	.516
No	151 (74.0)	53 (76.8)	98 (72.6)	

***Note***:

^†^Fisher’s Exact test.

### Frequently used antimicrobials among farmers

The preference and frequency of AMU in broiler and layer farms can be seen in Table 7, and both broiler and layer farmers listed frequently used antimicrobials. Both types of farmers utilized the mentioned antimicrobials almost equally, with the most frequently used antimicrobials being Colistin (broiler vs layer: 73.9% vs. 86.75%; *p* = 0.024) and Ciprofloxacin (broiler vs layer: 95.7% vs. 84.4%; *p* = 0.021).

**Table 7:** Pathways of the transmission of antimicrobial-resistant pathogens from poultry to humans.

Variables	Overall N = 204	Broiler	Layer	
	*n* (%)	n (%)	n (%)	
**Contaminated poultry products** *				
Contaminated meat	102 (50.0)	40 (58.0)	62 (45.9)	.104
Contaminated eggs	36 (17.6)	2 (2.9)	34 (25.2)	**<0.001** ^†^
I don’t know	78 (38.2)	26 (37.7)	52 (38.5)	.907
**Occurrence of direct/indirect contact**				
Humans with contaminated poultry	74 (36.3)	30 (43.5)	44 (32.6)	.229
Humans with contaminated fomite	63 (30.9)	21 (30.4)	42 (31.1)	
I don’t know	67 (32.8)	18 (26.1)	49 (36.3)	
**The appearance of waste material in the environment**				
Discharged contaminated litter	43 (21.1)	17 (24.6)	26 (19.3)	.524
Aerosols from poultry facilities	54 (26.5)	17 (24.6)	37 (27.4)	
Flies attracted to the contaminated litter	81 (39.7)	29 (42.0)	52 (38.5)	
I don’t know	26 (12.7)	6 (8.7)	20 (14.8)	

***Note***:

^†^Fisher’s Exact test;

*multiple responses.

### Pathways of the transmission of antimicrobial-resistant pathogens from poultry to humans

The pathways of the transmission of antimicrobial-resistant pathogens from poultry to humans are shown in Table 7. Far fewer broiler (2.9%, n = 2) compared to layer farmers (25.2%, n = 34) described contaminated poultry eggs as a source of transmission of antimicrobial-resistant pathogens from poultry to humans (*p* < 0.001).

## Discussion

Our study revealed that the use of antimicrobials is quite common in the poultry sector, with almost all broiler and layer poultry farmers administering antimicrobials to their chickens. The current study assessed the knowledge, attitudes, and practices of broiler and layer poultry farmers towards AMU. The findings revealed overall lower knowledge among both broiler and layer poultry farmers about appropriate AMU, source of AMU information, antimicrobial residues and pathogens, and the effect of antimicrobial resistance in humans.

Lower AMU knowledge can lead to antimicrobial misuse in farms and chickens, resulting in the growth of resistant pathogens. AMR has become a global issue in the last two decades, posing a serious threat to human and animal health [34,35]. Increased awareness through mass media, particularly television, and the constant repetition of essential messages might significantly reduce antibiotic abuse and the resulting AMR rates [36].

Our study explored farmers’ perspectives on different issues of AMU, where the majority of farmers reported desirable attitudes towards the use of antibiotics in chickens. This rate was higher among farmers who have achieved higher levels of education, agreeing with prior studies [33,37–41]. Due to a higher level of education which includes training and learning processes, farmers may become more aware of and have more access to veterinary services, farm management, and biosecurity measures, as well as a better understanding of the use of antimicrobials and their dose withdrawal periods [42]. A higher level of education, as well as farmers’ behaviors, are critical in the use of antimicrobials [33,37]. Farmers in Bangladesh with a poor level of knowledge (less than a 12th-grade education) were shown to depend on drug and feed dealers, neighboring farmers, and their own experiences, raising the risk of antibiotic abuse and the development of AMR [43]. Regarding practices of AMU, our study found that almost 90% of broiler and layer poultry farmers were using antibiotics on their farms. These results are comparable to other studies that have shown the high utilization of antibiotics to prevent infection in poultry farming [44–47]. The majority of farmers used antimicrobials for both therapeutic and prophylactic purposes. This finding is similar to other studies that reported the prophylactic use of antimicrobials to prevent frequently occurring poultry diseases [11] because of a lack of vaccination. Inadequate AMU laws and farmers’ lack of understanding of good practices of AMU may underlie the lack of control of these practices. The possibility of the development of AMR pathogens from these activities is determined by several parameters, most of which are related to the antimicrobials themselves, such as the quantity, dose, frequency, and duration of administration [48].

The antibiotic prescription pattern found in this study indicated that the majority of farmers purchased antibiotics from veterinary shops using a prescription. Our findings showed a discrepancy with other studies [49,50] where self-medication is common among poultry farmers due to farmers’ claims of good experience, the lack of veterinary services, and the higher cost of veterinary services. Our study found equal practices of administering antimicrobials to chickens either by animal health officers or by poultry farmers, which are inconsistent with other studies where self-administration of antimicrobials can result in under-dosing or over-dosing in poultry resulting in AM abuse [51]. Farmers in this study reported giving a daily single dose of antimicrobials to sick chickens until they had recovered, although it is just as crucial to utilize the right dose of antimicrobials as it is to fulfill the antimicrobial course [52].

Our study found that several antimicrobials were administered either alone or combined with other antimicrobials to treat various diseases, and farmers interviewed tended to use these antibiotics with drinking water. These findings are similar to other studies where drinking water was the preferred method for antibiotic treatment in chickens [44].

Our study found that the majority of farmers observed the recommended antimicrobial withdrawal period. These findings are inconsistent with the observation of other studies. Studies have found that the majority of Bangladeshi poultry farmers are unaware of the antibiotic withdrawal period [25,51]. Noncompliance with the appropriate withdrawal times may result in the presence of antibiotic residues in animal foods [53]. Antibiotic residues are potentially harmful to people and may contribute to the increase of AMR [53,54].

The findings of the study also showed that the majority of farmers stopped using antimicrobials before completing the full dose and, they did not keep records of antimicrobials that were used on their farms. While antimicrobial residues have previously been investigated in Bangladeshi poultry [55–57], rigorous efforts to screen for drug residue in marketed animal products are too restricted.

An interesting finding of this study was that several antimicrobials were most frequently administered either alone or in a mix with other antimicrobials. These findings are similar to prior studies that reported antimicrobials were frequently used in poultry production systems not only in Bangladesh but also in other countries in the world [29,32,58]. These antimicrobials, which include Colistin, Ciprofloxacin, and Tylosin, are frequently used in the poultry sector and are classified as “Critically Important Antimicrobials” for public health [59]. Bangladesh’s government has prohibited the use of antimicrobials in animal feed, including Colistin, for the manufacture of safe animal products [60,61]. However, their residues in chicken products can be passed on to humans, resulting in AMR manifestations.

In our study, farmers could identify the fundamental routes for the development of antibiotic-resistant pathogens and their transmission from poultry to humans. The following activities were most likely: infected poultry products (meat and eggs), direct or indirect interactions of poultry farmers with chickens and releases of waste material in the environment, including flies attracted to the contaminated litter. These findings are consistent with other studies that revealed resistant bacteria being passed from animals’ food to people through food intake, direct contact with infected animals, and animal waste in the ecosystem [18].

### Limitations

The present study has several limitations which need to be taken into consideration. Self-reported data might have influenced the results through the method, social desirability, and memory recall biases. The cross-sectional nature of the study means that no conclusions can be drawn regarding causality. Furthermore, our study only collected data during the production period and antimicrobials used in that production period; however, half of the farmers did not keep records and, one-third of the farmers did not observe the withdrawal periods of antimicrobials that they used in their farms. The study is also limited by the relatively small sample size and study participants within the Rajshahi district. So, generalization to the whole country is highly limited. Future studies need to overcome such limitations by employing longitudinal designs with larger and more representative samples.

## Conclusions

Our study indicated the majority of farmers had inadequate knowledge of appropriate AMU and less knowledgeable beliefs about AMU in chicken production systems. Half of the farmers surveyed did not keep records on AMU, and most did not complete the full dosage of antimicrobials. We recommend that steps are taken to ensure that farmers maintain the full dosage of antimicrobials that they use on their chickens, keep records of AMU (dose and duration of administration), as well as the usage of specific antimicrobials used. Special attention needs to be given to increasing awareness programs and educational interventions among poultry farmers. Furthermore, strategies to increase the efficiency of AMU research, monitoring, preventive, and control systems to ensure food safety, security, and public health.

## Supporting information

S1 File Data set. (XLSX)
